# Brain metastasis from differentiated thyroid carcinoma responding to radioiodine therapy

**DOI:** 10.22038/aojnmb.2025.86276.1618

**Published:** 2025

**Authors:** Leo Hashimoto, Shiro Watanabe, Mungunkhuyag Majigsuren, Kenji Hirata, Junki Takenaka, Rina Kimura, Hiroshi Ishii, Kohsuke Kudo

**Affiliations:** 1 Department of Nuclear Medicine, Hokkaido University Hospital, Sapporo, Japan; 2 Department of Diagnostic and Interventional Radiology, Hokkaido University Hospital, Sapporo, Japan; 3 Department of Diagnostic Imaging, Faculty of Medicine, Hokkaido University, Sapporo, Japan; 4 Global Center for Biomedical Science and Engineering, Faculty of Medicine, Hokkaido University, Sapporo, Japan; 5 Department of Radiology, Diagnostic Imaging Center, Second State Central Hospital, Ulaanbaatar, Mongolia; 6 Division of Medical AI Education and Research, Faculty of Medicine, Hokkaido University, Sapporo, Japan

**Keywords:** Thyroid cancer, Radioiodine, Brain metastasis

## Abstract

Brain metastasis (BM) occurs only in about 1% of differentiated thyroid carcinoma (DTC) cases. Although DTC generally has a good prognosis, once BM develops, the mortality rate significantly increases up to 78%. BM is usually treated by surgical resection or external radiotherapy, whereas radioactive iodine therapy (RAIT) using I-131 is much less often chosen because BM often shows poor uptake of I-131. In addition, even in case I-131 accumulates in the BM, RAIT could cause adverse effects such as brain hemorrhage and cerebral edema.

We present a case of BM from DTC that showed response to I-131 therapy with no severe adverse effects. The brain lesion was very small and asymptomatic, and was only found after a post-therapy I-131 scintigraphy. There are a few case reports where BM was cured by RAIT with little to no side effects. We theorize that BM that is small in size, asymptomatic and show I-131 accumulation could be successfully treated with RAIT.

## Introduction

 Brain metastasis (BM) occurs only in about 1% of differentiated thyroid carcinoma (DTC) cases (1). Although DTC generally has a good prognosis, once BM develops, the mortality rate significantly increases up to 78% (2). BM is usually treated by surgical resection or external radiotherapy, whereas radioactive iodine therapy (RAIT) using I-131 is much less often chosen because BM often shows poor uptake of I-131 (3). In addition, even in case I-131 accumulates in the BM, RAIT could cause adverse effects such as brain hemorrhage and cerebral edema (4).

 Here, we present a case of BM of DTC that showed response to I-131 therapy with no evident adverse effects.

## Case Reports

 A Japanese woman in her 40s was incidentally found to have multiple nodules in both lungs during a CT scan performed by a previous physician. The origin of these nodules was initially unclear, and a middle lobe resection was conducted to determine the nature of the lesions. Histopathological examination revealed that the nodules were metastatic lesions from thyroid carcinoma. Subsequent total thyroidectomy was performed, which confirmed the diagnosis of follicular variant papillary thyroid carcinoma, classified as T1bN0M1 (stage II). Given the diagnosis and the need for further treatment, the patient was referred to our department for consideration of radioactive iodine therapy.

**Figure 1:**
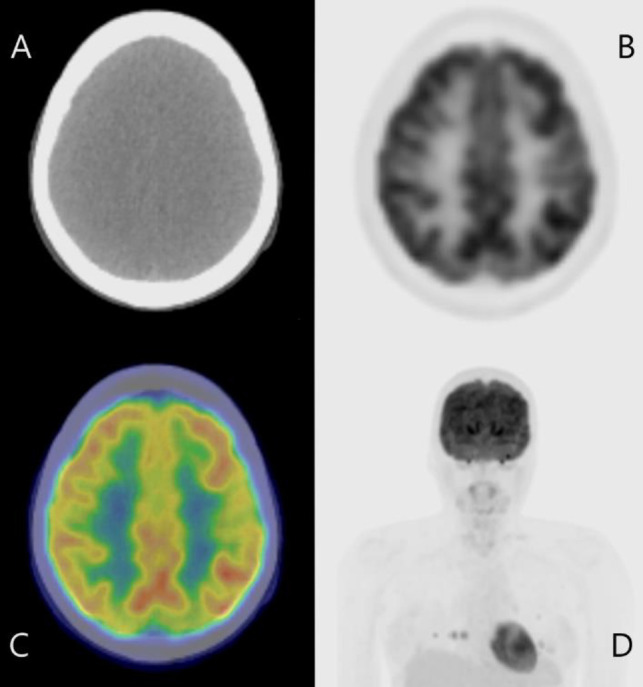
FDG-PET before radioactive iodine therapy (RAIT) showed no intracranial accumulation (**A**: CT for attenuation correction, **B**: FDG-PET (SUV range: 0-12), **C**: fusion image of CT and FDG-PET, **D**: anterior image of maximum intensity projection of FDG PET)

**Figure 2:**
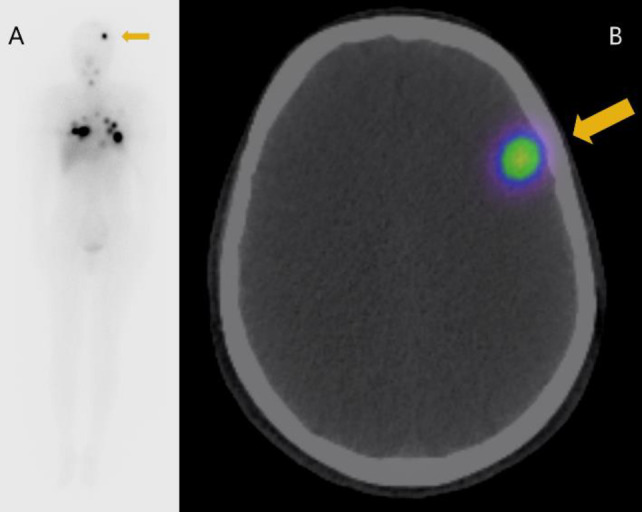
I-131 scintigraphy (**A**) and SPECT/CT (**B**) after the 1st radioactive iodine therapy (RAIT) showed accumulation in the left frontal lobe

**Figure 3:**
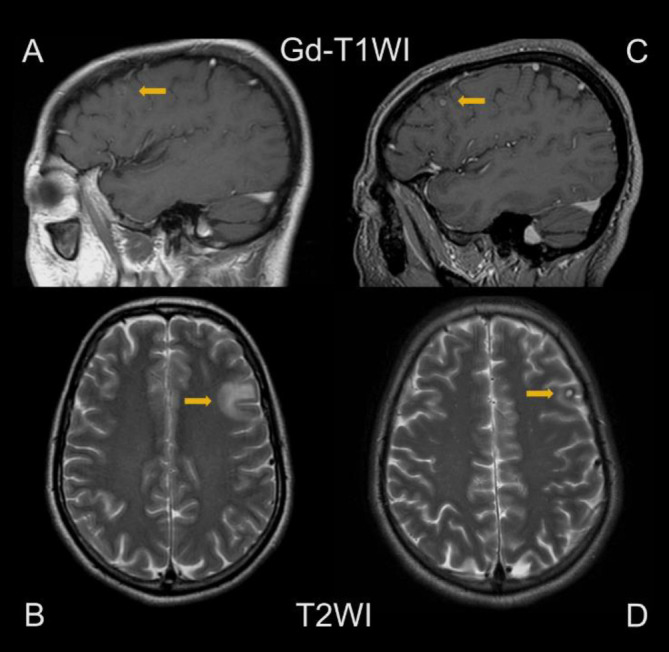
Brain MRI 1 month after the 1st radioactive iodine therapy (RAIT) (**A**, **B**) showed brain metastasis (BM) (8 mm in size) and peritumoral edema. Brain MRI 2 months after the 1st RAIT (**C**, **D**) showed shrinkage of BM and edema

FDG PET/CT scans before treatment showed no abnormal findings in the brain (Figure 1). Following a three-week period of iodine restriction and cessation of levothyroxine, the patient received a therapeutic dose of 150 mCi (5.55 GBq) of I-131. Post-therapy I-131 scintigraphy indicated uptake in multiple pulmonary metastases and an unexpected uptake in the left frontal lobe (Figure 2). A contrast-enhanced brain MRI performed one month after I-131 treatment showed an 8 mm ring-enhancing lesion in the left frontal lobe, suggestive of brain metastasis. T2-weighted imaging indicated a high-signal area surrounding the tumor, suggestive of edema (Figure 3A, B). Second MRI performed one month later revealed that the left frontal lobe metastasis had shrunk to 4 mm, with a reduction in the surrounding edema (Figure 3C, D).

**Figure 4:**
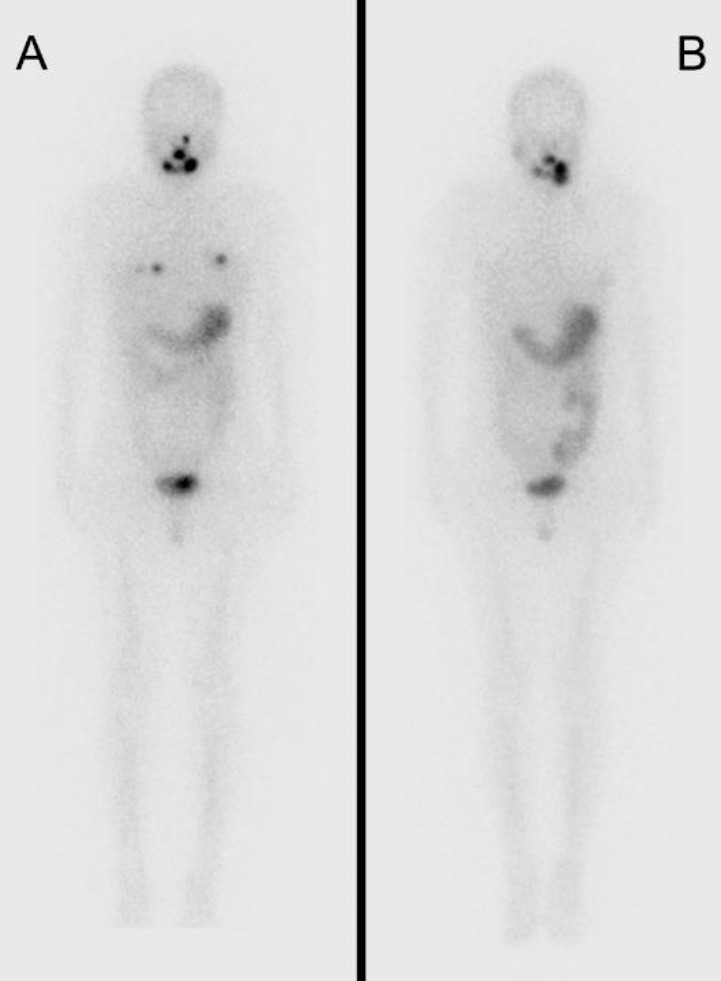
In the I-131 scintigraphy after the 2nd radioactive iodine therapy (RAIT) (**A**), accumulations have disappeared in the brain and shrunk in other regions. After the 3rd RAIT (**B**), accumulations have disappeared in all the region

 Two months after the first RAIT (following the second MRI evaluation), the patient underwent stereotactic radiotherapy with 32 Gy (in 4 fractions) for the brain metastasis. Six months after the initial I-131 therapy, a second round of I-131 treatment was administered. Post-therapy scintigraphy showed no uptake in the brain metastasis and reduced uptake in the pulmonary metastases (Figure 4A). One year later, the patient received a third round of I-131

therapy. Pre-therapy FDG-PET/CT showed complete remission of metastatic lesions. Post-therapy scintigraphy demonstrated the resolution of abnormal uptake in all previously identified metastases (Figure 4B) and the patient achieved an excellent response (Table 1) according to the 2015 American Thyroid Association guideline (5). The patient has since remained disease-free without recurrence.

**Table 1:** Results of blood tests before each radioactive iodine therapy (RAIT)

	1st RAIT	2nd RAIT	3rd RAIT
TSH (mIU/L)	27.45	130.94	155.36
Tg (ng/mL)	244.00	1.63	0.42
TgAb (IU/mL)	<3.00	<3.00	<3.00

**Tg:** Thyroglobulin, **TgAb:** Thyroglobulin Antibody, **TSH:** Thyroid-Stimulating Hormone

## Discussion

In the present case of BM of DTC, a brain lesion was incidentally discovered in post RAIT scintigraphy. It showed high I-131 uptake and responded well to RAIT. Although we observed mild peritumoral edema, no apparent neurological symptoms occurred throughout the pre- and post-treatment period.

 BM of DTC is extremely rare. It is also a significant adverse prognostic factor of DTC, which otherwise has a favorable prognosis. Surgical resection and external radiotherapy are recommended for the treatment of BM. On the other hand, RAIT is usually not chosen because it frequently causes hemorrhage from the lesion and edema in the surrounding brain (4). Additionally, pre-RAIT cessation of levo-thyroxine could also lead to peritumoral edema because the elevated serum thyroid-stimulating hormone (TSH) level causes tumor growth (6). 

 When patients have metastases in multiple organs in addition to BM, BM is usually treated first using aforementioned methods and then RAIT for other lesions. However, when surgery or external radiation are not feasible, RAIT is sometimes chosen as an alternative treatment. In these cases, glucocorticoid therapy is administered beforehand to prevent the swelling of BM (7).

 Despite BM from DTC having poor compatibility with RAIT, our case responded well to the treatment and showed no evident side effects. To evaluate the potential of RAIT as a primary treatment, we reviewed previous reports in which BM was treated with RAIT alone and showed good response without additional surgery or external radiation. As far as we could search, there are two such cases. In the first case, a 15-year-old Caucasian girl was found to have multiple lung, bone and brain metastases from papillary variant of thyroid carcinoma (8). There were two asymptomatic BM (up to 5mm in size), located in each frontal lobe. In consideration of the patient’s age, the small size of the lesions and their iodine avidity, RAIT under steroid cover was chosen as a primary treatment, and no prior radiotherapy or surgery was performed on BM. A series of RAIT with a total activity of 946 mCi (35 GBq) was administered. Without any side effects, a complete remission of all metastatic sites was achieved. She remained disease-free during the 

7.5-year follow-up period and the Tg level continued to decline to below 2 ng/mL. No late sequelae, including infertility or leukemia were observed. The second report presents a case of multiple lung and bone metastases, as well as a BM and a renal metastasis of thyroid carcinoma of indeterminate histology in a 65-year-old (presumably Asian) man presenting with chronic cough and expectoration (9). Similar to the previous case, this BM was iodine-avid, small and asymptomatic. The patient was administered RAIT under steroid cover and his respiratory symptoms subsided. Although the report follows the case only up to 2 months after the therapy, it is noteworthy that no notable side effects were observed during and after the therapy.

 When we compare our case with these two cases, one common feature is its smaller size. We theorize that, even if previously mentioned adverse effects happen, the small size of the BM contributed to their low severity. Additionally, the histological subtype of thyroid carcinoma can influence the response to RAIT, as RAI accumulation is known to vary between different histologies (10). Our case was follicular variant papillary thyroid carcinoma.

 Due to the rarity of BM from DTC, effectiveness of RAIT against it remains unclear. We propose that under certain circumstances, RAIT could be an effective and less invasive treatment compared to surgical resection and external radiation. Further studies are needed.

## Conclusion

 BM of DTC is seldom treated with RAIT because of its generally poor iodine uptake and possible severe adverse effects of the therapy. However, asymptomatic and smaller-sized (e.g., less than 10 mm) BM could potentially be treated safely with RAIT. Furthermore, RAIT could be a less invasive treatment option compared to surgical resection and whole-brain radiation therapy.
